# Defects in Halide Perovskites: Does It Help to Switch from 3D to 2D?

**DOI:** 10.1021/acsenergylett.4c00702

**Published:** 2024-04-23

**Authors:** Haibo Xue, Zehua Chen, Shuxia Tao, Geert Brocks

**Affiliations:** † Materials Simulation & Modelling, Department of Applied Physics, Eindhoven University of Technology, P.O. Box 513, 5600MB Eindhoven, The Netherlands; ‡ Center for Computational Energy Research, Department of Applied Physics, Eindhoven University of Technology, P.O. Box 513, 5600MB Eindhoven, The Netherlands; § Computational Chemical Physics, Faculty of Science and Technology and MESA+ Institute for Nanotechnology, University of Twente, P.O. Box 217, 7500AE Enschede, The Netherlands

## Abstract

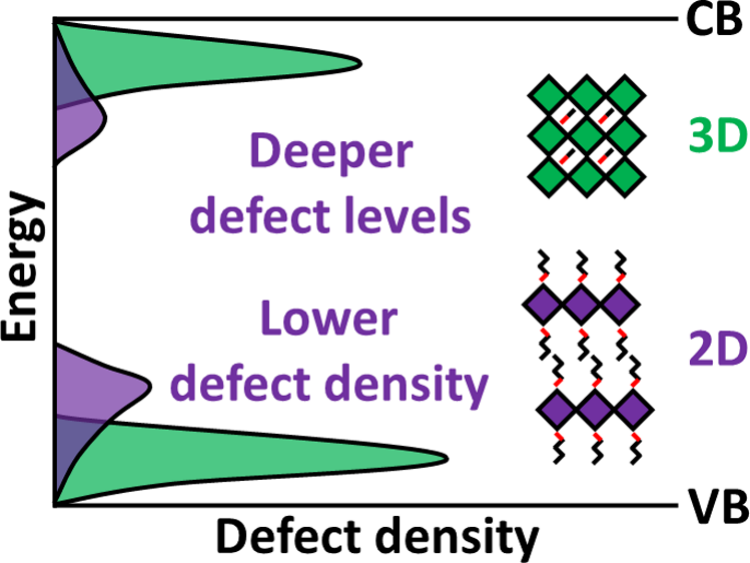

Two-dimensional (2D) organic–inorganic hybrid iodide perovskites have been put forward in recent years as stable alternatives to their three-dimensional (3D) counterparts. Using first-principles calculations, we demonstrate that equilibrium concentrations of point defects in the 2D perovskites PEA_2_PbI_4_, BA_2_PbI_4_, and PEA_2_SnI_4_ (PEA, phenethylammonium; BA, butylammonium) are much lower than in comparable 3D perovskites. Bonding disruptions by defects are more destructive in 2D than in 3D networks, making defect formation energetically more costly. The stability of 2D Sn iodide perovskites can be further enhanced by alloying with Pb. Should, however, point defects emerge in sizable concentrations as a result of nonequilibrium growth conditions, for instance, then those defects likely hamper the optoelectronic performance of the 2D perovskites, as they introduce deep traps. We suggest that trap levels are responsible for the broad sub-bandgap emission in 2D perovskites observed in experiments.

Hybrid organometal halide perovskites are materializing as candidate semiconductors for new generations of optoelectronic devices such as solar cells and light-emitting diodes.^1,2^ Application of these materials, however, is severely hampered by their lack of long-term stability.^3−6^ One of the most frequently studied compounds, MAPbI_3_, has favorable optical and charge transport properties,^7−10^ but the MA^+^ (methylammonium) ion is chemically not sufficiently stable and suffers from degradation reactions.^11,12^ Replacing MA^+^ by larger and more stable cations, such as FA^+^ (formamidinium)^13^ or GA^+^ (guanidinium),^14^ suffers from the perovskite structure becoming unstable, leading to a tendency to convert to different crystal structures that are much less optically active.^15−17^ This tendency can be suppressed to a certain extent by mixing in smaller inorganic cations, such as Cs^+^,^18,19^ but the fundamental issue remains that a stable 3D perovskite lattice requires the sizes of the constituting ions to be of a certain proportion, as expressed by the Goldschmidt tolerance factor,^2,20^ and the scale is set by the 3D network of metal halide octahedra in the perovskite.

In recent years, organometal halide perovskites with Ruddlesden–Popper or Dion–Jacobsen structures have emerged as alternative materials.^21^ In these perovskites the metal halide octahedra form a planar 2D network, and these 2D layers are separated by layers of organic cations, where the interlayer interaction is typically van der Waals.^22^ Using organic ions with a quasi-linear structure, such as PEA (phenethylammonium)^23^ or BA (butylammonium),^24^ the in-plane tolerance factor for a stable crystal structure is easily obeyed, whereas the out-of-plane size of the organic ion becomes relatively unimportant. Although the stability of such 2D perovskites is markedly improved, as compared to their 3D counterparts, presently photoelectric devices based upon 2D perovskites fail to reach the high efficiencies obtained with 3D perovskites.^22^ Lattice defects can play an important role in decreasing optoelectronic efficiencies. Previous studies have reported that iodine vacancies introduce deep traps in the band gap, responsible for the nonradiative recombination of charge carriers, as well as for the broad emission observed in 2D perovskites.^25−27^ Such findings indicate that 2D perovskites may lack the defect tolerance exhibited by 3D perovskites. However, it remains unclear what the thermodynamic trends of defects would be when switching from 3D to 2D perovskites, including knowledge of their structures and equilibrium concentrations. It also merits investigation to determine whether the compositional engineering could help in defect control for 2D perovskites.

In this paper, we explore the defect chemistry and physics of prominent 2D organometal iodide perovskites, PEA_2_PbI_4_, BA_2_PbI_4_, and PEA_2_SnI_4_, and of the alloy PEA_2_Sn_0.5_Pb_0.5_I_4_, using first-principles density functional theory (DFT) calculations. The ease with which point defects can be created in a material is an indication for its stability. We therefore focus on the defect formation energy (DFE), as it can be calculated assuming thermodynamic equilibrium conditions. We use the same formalism applied in our previous work on 3D perovskites.^28^ A summary of the theory is given in Section S1 in the Supporting Information (SI). The equilibrium chemical potentials of the different elements are determined by considering the phase diagram of the 2D perovskite; see Figure S1 in the SI. The defect formation energies are calculated using iodine-medium conditions, which are the conditions most typically relevant.

Even if defects do not occur in large quantities under thermodynamic equilibrium conditions, they may appear more prominently under nonequilibrium growth conditions, or under operating conditions.^29^ If so, they can seriously affect the electronic properties of the material, as defect states with energy levels inside the semiconductor band gap can act as traps for charge carriers and as recombination centers for radiationless decay. We explore these energy levels, called charge-state transition levels (CSTLs), associated with the most likely point defects in the 2D materials listed above.

DFT calculations are performed with the Vienna *Ab Initio* Simulation Package (VASP),^30−32^ employing the SCAN+rVV10 functional^33^ for electronic calculations and geometry optimization. The SCAN+rVV10 functional is used, aiming at obtaining accurate defective structures and total energies, and therefore DFEs, which are the main focus of this work. Whereas the DFT band gap error may result in incorrect band edges, the calculated CSTLs are suggested to be correct in a relative sense, as discussed in our previous work.^34^

Structures of PEA_2_PbI_4_, BA_2_PbI_4_, and PEA_2_SnI_4_ are taken from experiments, see refs (35−37), respectively, and are then optimized, including the volume of the unit cell. A structure for PEA_2_Sn_0.5_Pb_0.5_I_4_ is constructed by substituting half of the Pb by Sn ions in PEA_2_PbI_4_, before reoptimizing the structure; see the Figure S2 in the SI. Detailed computational settings and structures are discussed in Section S1 in the SI, including the procedure used for creating defective structures.

We start with point defects in the most popular 2D perovskite, PEA_2_PbI_4_, i.e, the PEA vacancy V_PEA_, the Pb vacancy V_Pb_, and the iodine vacancy V_I_, and the interstitials Pb_i_ and I_i_. The PEA interstitial is omitted, because the structure is too dense to additionally accommodate an extra organic cation of such a large size. In addition to these simple point defects, we also study the compound vacancies V_PEAI_ and V_PbI_2__, representing missing units of the precursors PEAI and PbI_2_. The layered nature of the 2D perovskite, (Figure 1a), implies that iodine vacancies and interstitials in the central PbI planes and those outside these planes can behave differently, and both configurations are studied. Optimized structures of all defects in their most stable charge states are shown in Figure 1b–j.

**Figure 1:**
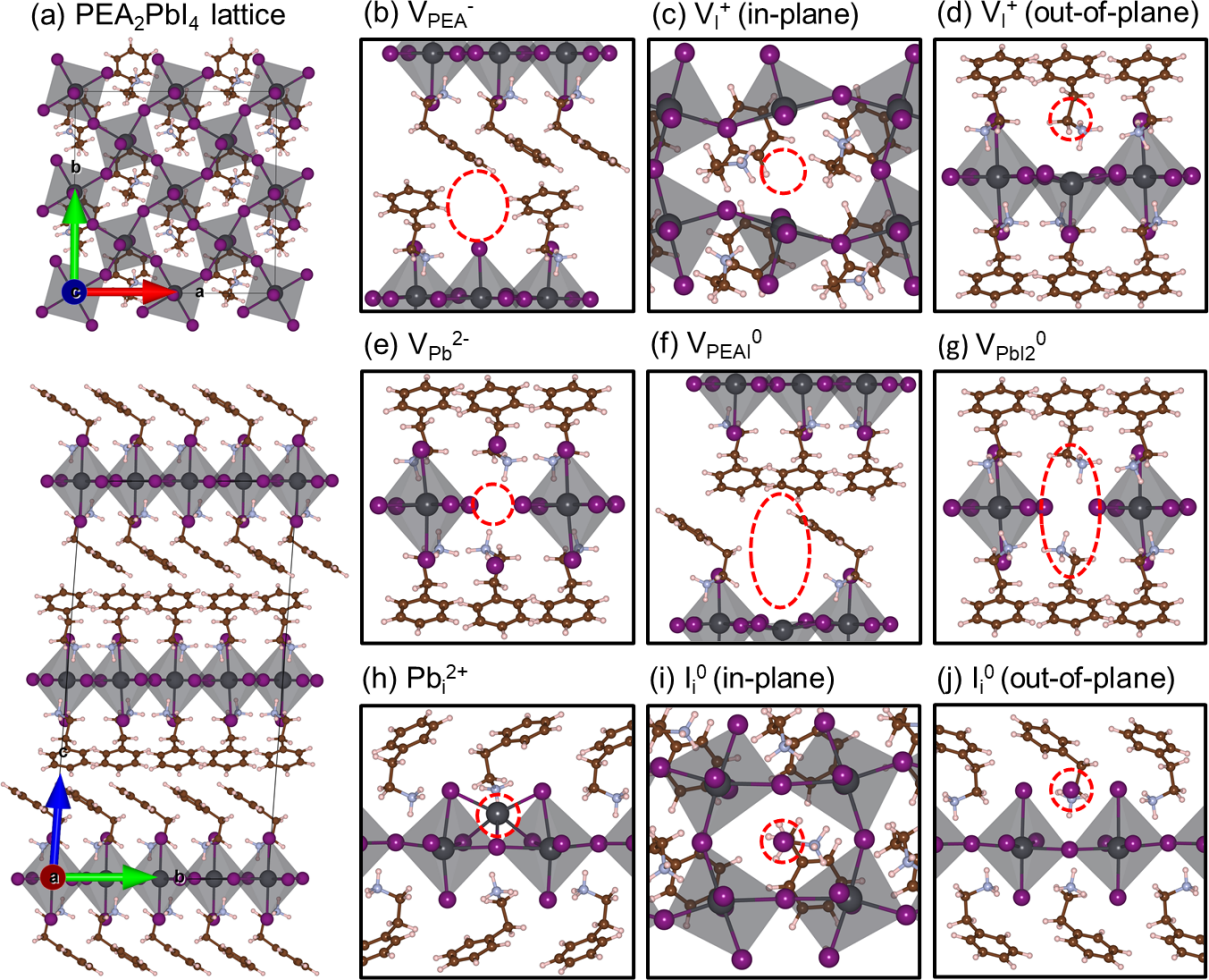
(a) Top and side views of a 2 × 2 × 1 PEA_2_PbI_4_ supercell; optimized structures of vacancies (b–g) and interstitials (h–j) in their most stable charge states in PEA_2_PbI_4_. The positions of the defects are marked in red. The labels (in-plane) and (out-of-plane) refer to positions of iodine vacancies and interstitials either within a PbI_2_ plane or above/below it.

The calculated DFEs of PEA_2_PbI_4_ are shown in Figure 2a. The intrinsic Fermi level (*E*_F_^(i)^ = 0.68 eV with respect to the valence band maximum, VBM) is obtained from the charge neutrality condition; see Section S1.3.3 in the SI. At this condition, the vacancies V_PEA_^–^ and V_Pb_^2–^ are easiest to form and thereby are the most dominant defects, with formation energies of 0.89 and 0.82 eV, respectively. This leads to equilibrium concentrations at room temperature of 1.29 × 10^7^ and 1.21 × 10^8^ cm^–3^, respectively. Other vacancies, V_I_^+^, and the compound vacancies V_PEAI_^0^ and V_PbI_2__^0^, as well as all interstitial species, have formation energies ≳1 eV and are thus unimportant under equilibrium conditions (with the intrinsic Fermi level). A full list of formation energies and concentrations at room temperature of defects is given in Table 1.

**Figure 2:**
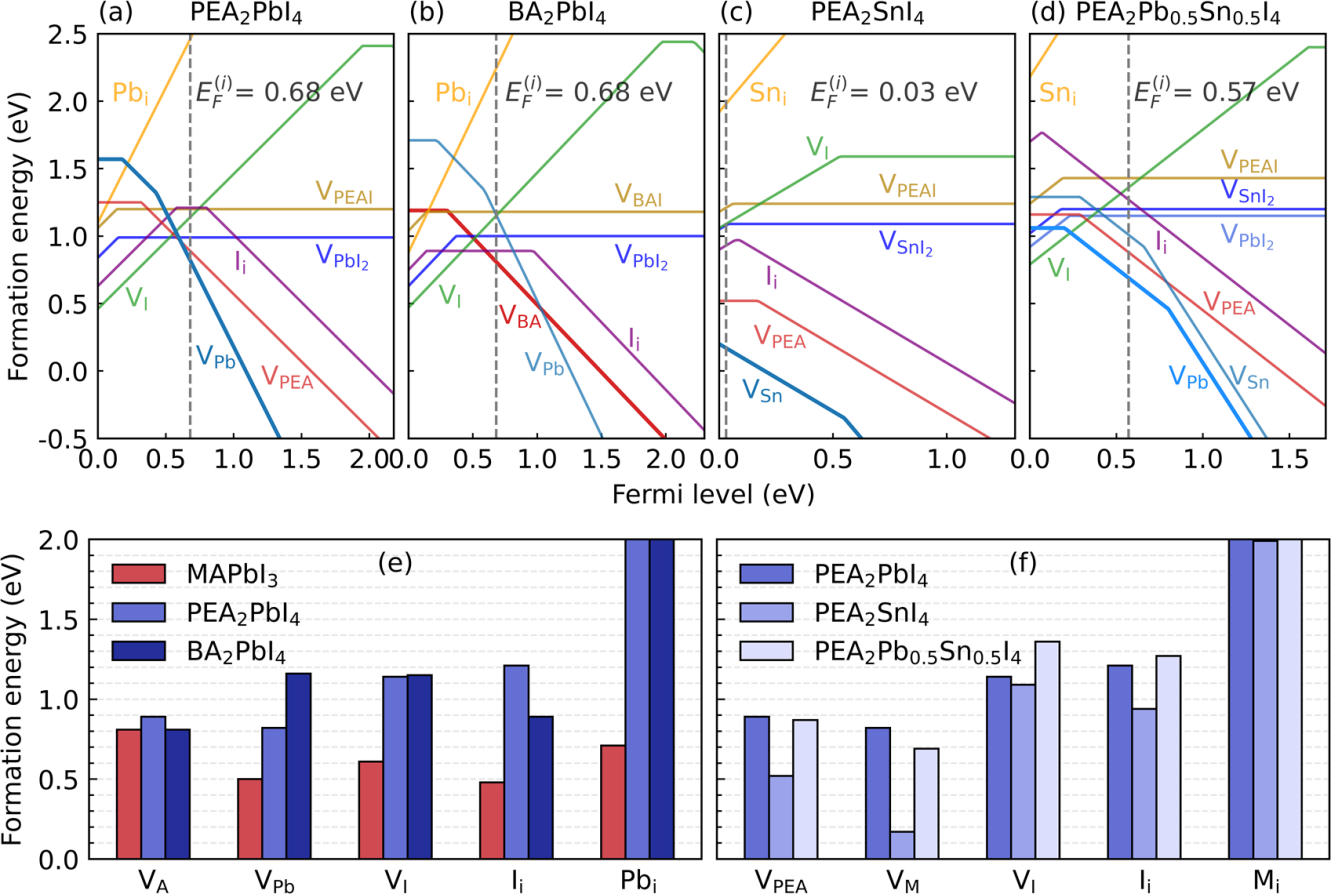
(a–d) Defect formation energies in PEA_2_PbI_4_, BA_2_PbI_4_, PEA_2_SnI_4_, and PEA_2_Pb_0.5_Sn_0.5_I_4_ as a function of the position of the Fermi level. The intrinsic Fermi level is indicated by the black dashed line; DFEs of these perovskites calculated at the I-rich and I-poor conditions are given in Figure S3 in the SI. Comparison of DFEs at the intrinsic Fermi level to MAPbI_3_ (e) and among 2D perovskites with different metal cations (f). The DFEs of Pb_i_ and Sn_i_ in 2D perovskites are larger than 2 eV, with the detailed values being listed in Table 1.

**Table 1:** Formation Energies Δ*H*_f_ (eV) and Concentrations *c* (cm^–3^) at *T* = 300 K of Defects in 2D Halide Perovskites in Their Most Stable Charge States, Calculated at the Intrinsic Fermi Level and Iodine-Medium Conditions, Using the SCAN+rVV10 Functional^a^

	V_A_^–^	V_M_^2–^	V_I_^+^	I_i_^–^	M_i_^2+^
Defect Formation Energy Δ*H*_f_ (eV)					
MAPbI_3_	0.81	0.50	0.61	0.48	0.71
PEA_2_PbI_4_	0.89	0.82	1.14	1.21 (I_i_^0^)	2.46
BA_2_PbI_4_	0.81	1.16	1.15	0.89 (I_i_^0^)	2.24
MASnI_3_ (Sn-rich)^b^	0.58	0.26	0.68	0.40	0.81
PEA_2_SnI_4_ (I-med)	0.52 (V_PEA_^0^)	0.17 (V_Sn_^–^)	1.09	0.94 (I_i_^+^)	1.99
PEA_2_SnI_4_ (Sn-rich)^b^	0.65	0.46 (V_Sn_^–^)	1.09	1.02 (I_i_^–^)	1.99
PEA_2_Pb_0.5_Sn_0.5_I_4_	0.87	0.69(V_Pb_^–^)	1.36	1.27	3.68 (Pb_i_^2+^)
		1.01 (V_Sn_^–^)			3.33 (Sn_i_^2+^)
Defect Concentration *c* (cm^–3^)					
MAPbI_3_	8.68 × 10^7^	1.44 × 10^13^	6.10 × 10^11^	1.37 × 10^14^	6.36 × 10^10^
PEA_2_PbI_4_	1.29 × 10^7^	1.21 × 10^8^	2.01 × 10^3^	1.58 × 10^2^	1.34 × 10^–19^
BA_2_PbI_4_	4.39 × 10^8^	2.70 × 10^2^	1.31 × 10^3^	3.23 × 10^7^	6.15 × 10^–16^
MASnI_3_ (Sn-rich)^b^	6.62 × 10^11^	1.60× 10^17^	4.18 × 10^10^	2.36 × 10^15^	1.09 × 10^9^
PEA_2_SnI_4_ (I-med)	2.51 × 10^13^	1.11 × 10^19^	1.13 × 10^4^	5.25 × 10^6^	9.51 × 10^–12^
PEA_2_SnI_4_ (Sn-rich)^b^	1.53 × 10^11^	1.27 × 10^14^	1.13 × 10^4^	1.90 × 10^5^	9.53 × 10^–12^
PEA_2_Pb_0.5_Sn_0.5_I_4_	2.98 × 10^7^	9.81 × 10^9^	4.13 × 10^–1^	4.64 × 10^0^	3.96 × 10^–40^
		4.06 × 10^4^			3.50 × 10^–34^

a For comparison, the corresponding numbers for 3D perovskites MAPbI_3_ and MASnI_3_ have been added. The formation energies and concentrations of the dominant defects in each perovskite are underlined. For defects that have a deviating most stable charge state at the intrinsic Fermi level, that state is specified in parentheses.

b To facilitate comparison to the other compounds, formation energies and defect concentrations in MASnI_3_ and PEA_2_SnI_4_ calculated under Sn-rich (I-poor) conditions are provided.

These findings are in stark contrast with results obtained for 3D perovskites, calculated using the same computational settings.^28^ In the archetype 3D perovskite MAPbI_3_, the formation energies of several point defects are ≲0.5 eV, which leads to equilibrium defect concentrations that are 6 orders of magnitude higher at room temperature than in PEA_2_PbI_4_^28^ (Figure 2e). Moreover, interstitials generally play a significant role in the defect chemistry of 3D perovskites.^28^ In MAPbI_3_, besides the vacancy V_Pb_^2–^, the dominant point defects are the interstitials MA_i_^+^ and I_i_^–^. By comparison, interstitials in PEA_2_PbI_4_ are unimportant relative to the vacancies.

The difference between interstitials in 2D and 3D perovskites is also expressed in their chemical bonding patterns. Iodine interstitials in 3D perovskites are inserted between two Pb atoms in the lattice, next to an already present iodine ion, forming a Pb–I_2_–Pb unit with two equivalent Pb–I–Pb bridge bonds.^28,38^ In contrast, iodine interstitials in PEA_2_PbI_4_ prefer to stay between two lattice iodines, forming a I_3_ trimer structure (Figure 1i,j). This difference in the bonding pattern is also reflected in the most stable charge state. Whereas the iodine interstitial in 3D perovskites is negatively charged, in PEA_2_PbI_4_ it is stable in the neutral state.

The more prominent defects in PEA_2_PbI_4_, the vacancies V_Pb_^2–^ and V_PEA_^–^ (Figure 1b,e), and also the less prominent vacancies V_I_^+^, V_PEAI_^0^ and V_PbI_2__^0^ (Figure 1c,d,f,g), have bonding patterns that are qualitatively similar to those in 3D perovskites, and stable charge states that are the same. The DFEs of these defects in PEA_2_PbI_4_ are significantly higher, though. Whereas in 3D perovskites, the presence of a vacancy can be accommodated to some extent by rearranging the lattice around the vacancy, in a 2D perovskite such a rearrangement is more difficult. In addition, as Coulomb interactions in 2D perovskites are less screened than in comparable 3D perovskites, because of the smaller dielectric constants of the former, one may expect these interactions to be stronger. This means that removing an ion, which creates a vacancy, might be more difficult in 2D perovskites.^39−41^

The analysis of results obtained for PEA_2_PbI_4_ also holds qualitatively for other 2D perovskites, such as BA_2_PbI_4_, whose DFEs are shown in Figure 2b. Using PEA_2_PbI_4_ as a reference, it is observed that the formation energies of defects in BA_2_PbI_4_ are 0.01–0.34 eV different from the corresponding ones in PEA_2_PbI_4_ (Figure 2e). The vacancy V_BA_^–^ has a somewhat smaller DFE than the vacancy V_PEA_^–^, 0.81 eV versus 0.89 eV, which likely reflects the fact that removing the smaller BA^+^ leaves a smaller hole in the lattice. As for the other defects, vacancies in BA_2_PbI_4_ tend to be somewhat destabilized, compared to those in PEA_2_PbI_4_, whereas interstitials are somewhat stabilized. None of these changes affect the qualitative comparison to 3D perovskites as discussed above, however.

Stabilization of compounds in 2D structures offers an interesting perspective for Sn-based perovskites, where, for instance, the 3D MASnI_3_ perovskite is quite unstable. That is reflected by the ease with which V_Sn_^2–^ vacancies are generated spontaneously (see Table 1), making this compound an intrinsically doped degenerate p-type semiconductor.^28^ In the literature this is related to the fact that Sn^2+^ can be easily oxidized to Sn^4+^.^42−45^ In fact, calculations indicate that MASnI_3_ is thermodynamically stable only under rather extreme iodine-poor conditions (Table 1).^28^

Figure 2c shows the formation energies of defects in PEA_2_SnI_4_, calculated under milder, iodine-medium, conditions. All DFEs are positive, indicating that the material is stable against the spontaneous formation of defects, which is in contrast with MASnI_3_ (see Table 1). The vacancy V_Sn_ in PEA_2_SnI_4_ is the defect with the lowest DFE, but the latter is considerably higher than the corresponding DFE in MASnI_3_, signaling an increased stability of the material. The vacancy is negatively charged, V_Sn_^–^, and, as is common in Sn-based halide perovskites, there is no appreciable concentration of positively charged defects to maintain charge neutrality.^28,46^ The latter has to be ensured by holes in the valence band, which leads to an intrinsic Fermi level that is only 0.03 eV above the VBM. This results in PEA_2_SnI_4_ being an intrinsic p-type semiconductor, albeit not a degenerate one, as is the case for MASnI_3_. This agrees with the experiments that Sn-based 2D perovskites are hole-doped materials, evidenced by the large hole concentration and hole mobility.^47−49^ The stability of PEA_2_SnI_4_ can be further improved by growing the material under the Sn-rich (or I-poor) growth condition (see Figure S3h,i). This holds true for both 2D and 3D Sn-based perovskites, as shown by the DFEs and defect concentrations of the Sn vacancies in Table 1. In experiments, this can be realized by adding the SnF_2_ additive to suppress the formation of Sn vacancies.^50−52^

The DFE of the main defect, V_Sn_^–^, in PEA_2_SnI_4_ is 0.17 eV, which gives an equilibrium concentration (at room temperature) of 1.11 × 10^19^ cm^–3^. The vacancy V_PEA_^0^ has a DFE of 0.52 eV and an equilibrium concentration of 2.51 × 10^13^ cm^–3^, whereas other vacancies and interstitials have a DFE in excess of 0.94 eV, so they do not play a significant role. Compared to the most stable charge states of the prominent defects in PEA_2_PbI_4_ (V_Pb_^2–^ and V_PEA_^–^), note that those in PEA_2_SnI_4_ are +1e higher (V_Sn_^–^ and V_PEA_^0^). This stems from the low-lying intrinsic Fermi level in PEA_2_SnI_4_, as compared to that in PEA_2_PbI_4_, consistent with an increased p-type doping (compare Figure 2a,c).

One can observe that the DFEs in PEA_2_SnI_4_ tend to be significantly smaller than the corresponding ones in PEA_2_PbI_4_ (see Figure 2f). The intrinsic defect concentrations in the former are therefore much higher, which is a clear sign of a decrease in the stability. In particular, 2D Sn-based perovskites inherit the problem of a large number of Sn vacancies from the 3D Sn-based perovskites. To suppress the formation of Sn vacancies, one solution is to mix Sn with Pb.^45,46^ We investigate its effect using perovskite PEA_2_Pb_0.5_Sn_0.5_I_4_. As an example, we study a highly ordered structure of PEA_2_Pb_0.5_Sn_0.5_I_4_, whose construction is discussed in Figure S2 of the SI. The calculated DFEs are shown in Figure 2d. It is evident that it is more difficult to form defects in PEA_2_Pb_0.5_Sn_0.5_I_4_ as compared with the pure Sn-based perovskite PEA_2_SnI_4_. In addition, the intrinsic Fermi level in PEA_2_Pb_0.5_Sn_0.5_I_4_ is calculated at 0.57 eV, making it a mildly doped intrinsic p-type semiconductor, which, compared to PEA_2_SnI_4_, is more in line with the other 2D perovskites (see Figure 2a–d).

The defect in PEA_2_Pb_0.5_Sn_0.5_I_4_ that is easiest to form is the vacancy V_Pb_^–^ with a DFE of 0.69 eV, whereas the DFE of V_Sn_^–^ is 0.32 eV higher. Although this particular difference may be the result of the particular ordered structure we have chosen to represent PEA_2_Pb_0.5_Sn_0.5_I_4_, we would argue that DFEs of metal vacancies in this compound are larger than those in pure PEA_2_SnI_4_, provided Sn and Pb metal atoms are well mixed on an atomic scale. That this is the case is corroborated by optical measurements.^53^ The DFEs of other defects, such as V_PEA_^–^, are comparable to those in pure PEA_2_PbI_4_, or even higher (see Figure 2f). In summary, mixing Pb and Sn in the 2D perovskite significantly suppresses the formation of defects compared to the pure Sn-based perovskite and maintains the defect tolerance of the pure Pb-based perovskite.

Although equilibrium defect concentrations in the 2D perovskites considered here, with the exception of PEA_2_SnI_4_, are predicted to be small, materials are often grown under highly nonequilibrium conditions, during which a substantial amount of defects can form.^29,54^ Likewise, under solar cell operating conditions, the (quasi) Fermi levels are very different from the intrinsic Fermi level, which may stimulate the formation of certain defects, as shown in Figure 2. These two points motivate investigating whether defects lead to electronic levels inside the band gap of a 2D material that can be harmful to its electronic operation.

The charge state transition levels (CSTLs) are shown in Figure 3 for each point defect and compound vacancy in each of the 2D perovskites studied here. Generally, CSTLs are considered to be deep levels when their energy distance from the band edges is much larger than the thermal energy *k*_B_*T* (0.026 eV at the room temperature).^55^ Deep levels can trap charge carriers and cause significant (nonradiative) recombination, thereby reducing solar cell or light-emitting diode efficiencies. What can be observed in Figure 3 is that most point defects in 2D perovskites lead to deep levels. In fact, only the compound vacancies, V_PEAI_ and V_PbI_2__, give shallow (acceptor) levels. This is in remarkable contrast to what is found for 3D perovskites, where many point and compound defects produce shallow levels.^28,29^ This immediately provides a possible explanation of why the efficiency of 2D halide perovskite solar cells is generally lower than those based on 3D perovskites.

**Figure 3:**
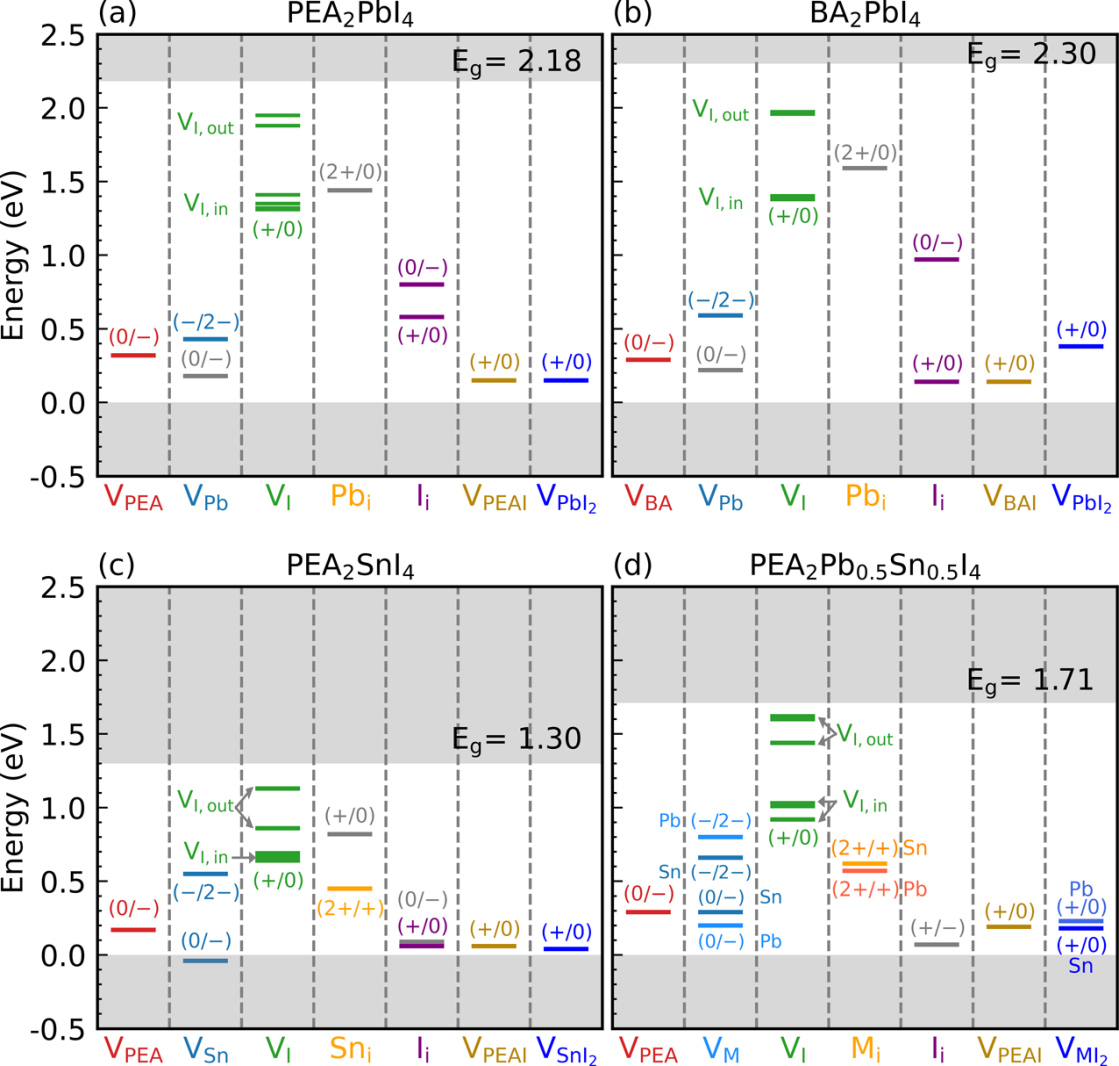
Charge state transition levels (CSTLs) of defects in PEA_2_PbI_4_ (a), BA_2_PbI_4_ (b), PEA_2_SnI_4_ (c), and PEA_2_Pb_0.5_Sn_0.5_I_4_ (d). The most important ones are indicated by colored lines, representing a change of a single unit ±*e* starting from one stable charge state of a defect; the bottom and top gray areas represent the valence and conduction bands (calculated with SCAN+rVV10), aligned at the VBM. In (d), M represents metal cations, Pb and Sn.

For 3D perovskites it is argued that many defects producing shallow levels stem from the fact that defect states in these materials have either valence band or conduction band character, depending on the type of defect, and that the disruption in the chemical bonding pattern caused by the defect is not so large as to move the defect levels in energy far from the band edges. Figure 3 shows that the CSTLs in 2D perovskites are still either in the top half or in the bottom half of the band gap, depending on the type of defect, but apparently the disruption caused by the defects is now sufficiently large to move defect levels well inside the band gap. In addition, as the dielectric constant of 2D perovskites (Table S2 in the SI) tends to be smaller than that of their 3D counterparts,^56,57^ the Coulomb interaction is stronger, which also contributes to defect levels being deeper.

As an example, consider the organic cation vacancies in 3D perovskites such as MAPbI_3_, FAPbI_3_, or MASnI_3_, where V_MA_^–^ and V_FA_^–^ only give a very shallow acceptor level just above the VBM.^28^ In all 2D compounds studies here, V_PEA_^–^ or V_BA_^–^ gives an acceptor level ≳0.2 eV above the VBM that can act as a trap state (see the first columns in Figure 3a–d). Likewise, the Pb and Sn vacancies, V_Pb_^2–^ and V_Sn_^2–^, which only give very shallow acceptor levels in 3D perovskites,^28^ give a couple of deep trap levels in 2D perovskites, as can be observed from the second columns in Figure 3a–d.

An interesting case is the iodine vacancy V_I_, which in 3D perovskites typically only gives shallow acceptor levels. In 2D perovskites, V_I_ generates different levels according to the position of the vacancy, whether above/below or in a PbI_2_ plane (see Figure 1c,d). Vacancies associated with the in-plane positions generate deep levels that are 0.6–0.9 eV below the CBM, whereas iodine vacancies located outside the PbI_2_ planes give donor levels that are much closer to the conduction band edge.

In experiments, a broad emission of light with frequencies corresponding to the upper half of the band gap is frequently found in photoluminescence (PL) spectra of 2D perovskites, where the peak of the emission spectrum is approximately at 0.6 eV below the CBM.^26,58^ We suggest that this emission may be associated with defect states created by iodine vacancies, should they form in sizable concentrations under nonequilibrium growth conditions. This suggestion aligns with the finding that iodine vacancies at in-plane positions, with the CSTL (+/0) being 0.64 eV below the CBM, have a PL emission energy comparable to the experimental value.^27^ The only other defect that gives a level in the upper half of the band gap, the Pb interstitial Pb_i_, is not likely to occur in appreciable quantities, as shown in Figure 2. Moreover, the Sn interstitial, Sn_i_, only gives a level close to the VBM, and the broad emission of the type discussed above is also observed in 2D Sn-based perovskites.

To conclude, we employ first-principles calculations to study the defect formation energies and charge state transition levels of intrinsic defects in hybrid organic–inorganic iodide 2D perovskites. We find that the equilibrium concentrations of point defects in the 2D perovskites PEA_2_PbI_4_, BA_2_PbI_4_, and PEA_2_SnI_4_ are much lower than those in comparable 3D perovskites, indicating an improved material stability of 2D perovskites. The stability of 2D Sn iodide perovskites can be further enhanced by alloying with Pb. Moreover, unlike the prominence of interstitials in 3D perovskites, 2D perovskites are dominated by vacancies. The difficulty in forming defects in 2D perovskites is attributed to two factors. One is that the bonding disruptions involved in creating defects are more destructive in 2D than in 3D networks. Another is that the dielectric constants are smaller for 2D perovskites, which makes Coulomb interactions larger, and removing ions more energetically costly. These factors also cause the formation of deep defect levels in the band gap of 2D perovskites. Consequently, should point defects emerge in sizable concentrations, then those defects can hamper the optoelectronic performance of the 2D perovskites. Finally, we suggest that the trap levels of iodine vacancies are responsible for the broad sub-bandgap emission in 2D perovskites observed in experiments.

## Supporting Information Available

Detailed computational approaches, including DFT calculations, structures, defect formation energy, chemical potential, finite-size correction, intrinsic Fermi level, and charge state transition level, structures of mixed Pb–Sn perovskite, and defect formation energies of each perovskite at different growth conditions (PDF)
