# Supplementary material for: Defects in Halide Perovskites: Does It Help to Switch from 3D to 2D?
Source: ACS Energy Lett. 2024 Apr 23;9(5):2343–50. doi: 10.1021/acsenergylett.4c00702 (PMC11091873; doi:10.1021/acsenergylett.4c00702)
Supplement: Supplementary file 1 — nz4c00702_si_001.pdf [file nz4c00702_si_001.pdf]

Supporting Information: Defects in Halide Perovskites: Does It Help to Switch from 3D to 2D?

Haibo Xue,[†,‡] Zehua Chen,[†,‡] Shuxia Tao,[*,†,‡] and Geert Brocks[*,†,‡,¶]

†*Materials Simulation & Modelling, Department of Applied Physics, Eindhoven University of Technology, P.O. Box 513, 5600MB Eindhoven, the Netherlands.*

‡*Center for Computational Energy Research, Department of Applied Physics, Eindhoven University of Technology, P.O. Box 513, 5600MB Eindhoven, the Netherlands.*

¶*Computational Materials Science, Faculty of Science and Technology and MESA+ Institute for Nanotechnology, University of Twente, P.O. Box 217, 7500AE Enschede, the Netherlands.*

E-mail: s.x.tao@tue.nl; g.h.l.a.brocks@utwente.nl

Contents

S1 Computational Approaches **S3**

1 DFT calculations S3

2 Structures S3

3 Defect formation energy S4

3.1 Chemical potential S5

3.2 Finite-size supercell correction and potential alignment S6

3.3 Intrinsic Fermi level S7

4 Charge state transition level S8

S2 Mixed Pb-Sn perovskite **S9**

S3 DFEs at different growth conditions **S10**

References **S11**

S1 Computational Approaches

1 DFT calculations

Density functional theory (DFT) calculations are performed with the Vienna *Ab-Initio* Simulation Package (VASP),[S1–S3] employing the SCAN+rVV10[S4] functional for electronic structure and geometry optimization. This functional combines the strongly constrained and appropriately normed (SCAN)[S5] meta-generalized gradient approximation (meta-GGA) functional with the long-range van der Waals interactions from the revised Vydrova-van Voorhis non-local correlation functional (rVV10).[S6] It has emerged as a reliable functional for calculating defect properties of metal halide perovskites in our previous work, Ref. S7.

Spin-orbit coupling (SOC) is omitted, as it has little effect on the formation energies of defects.[S7] Our calculations use a plane wave kinetic energy cutoff of 450 eV (see Table S1 for the convergence test), and a Γ-point only **k**-point mesh. The energy and force convergence criteria are set to 10^{-4} eV and 0.02 eV/Å, respectively. Spin-polarization is included in all calculations.

Table S1: Convergence test of the cutoff energy of the plane-wave-basis set using the PEA_2PbI_4 unitcell. For each cutoff energy, the lattice volume and ionic positions are fully relaxed.

Cutoff energy (eV)	Lattice constant (Å)		
	a	b	c
375	8.58	8.58	31.72
400	8.60	8.60	31.79
425	8.61	8.61	31.84
450	8.63	8.63	31.90
475	8.63	8.63	31.90
500	8.63	8.63	31.90

2 Structures

Structures of PEA_2PbI_4, BA_2PbI_4 and PEA_2SnI_4 are taken from the experimentally determined lattices, see Refs. S8–S10, respectively. $PEA_2Sn_{0.5}Pb_{0.5}I_4$ is constructed by substitut-

ing Pb with Sn in PEA_2PbI_4, see section S2 for the detailed discussion about the substitution strategy. The structures are then optimized using the SCAN+rVV10 functional, including optimizing the volume of the unit cell.

Defective structures are created starting from 2×2×1 supercells, which contain 16 formula units per supercell, with 752 and 624 atoms for PEA- and BA-based perovskites, respectively. An interstitial is created by adding to the supercell a cation or an anion in a specific charge state, and then optimize the atomic positions within the supercell. Likewise, a vacancy is created by removing from the supercell a cation or an anion.

3 Defect formation energy

The defect formation energy ΔH_f is calculated from the expression[S11]

$$\Delta H_f(D^q) = E_{\text{tot}}(D^q) - E_{\text{tot}}(\text{bulk}) - \sum_k n_i\ \mu_i + q(E_F + E_{\text{VBM}} + \Delta V) + E^q_{\text{corr}}, \tag{S1}$$

where $E_{\text{tot}}(D^q)$ and $E_{\text{tot}}(\text{bulk})$ are the DFT total energies of the defective and pristine supercells, respectively, and n_i and μ_i are the number of atoms and chemical potential of atomic species i added to ($n_i > 0$) or removed from ($n_i < 0$) the pristine supercell in order to create the defect.

Creating a charge q requires taking electrons from or adding them to a reservoir at a fixed Fermi level. The latter is calculated as $E_F + E_{\text{VBM}}$, with $0 \leq E_F \leq E_g$, the band gap, and E_{VBM} the energy of the valence band maximum. As it is difficult to determine the latter from a calculation on a defective cell, one establishes E_{VBM} in the pristine cell, shifted by ΔV, which can be obtained by aligning a core level or averaged potential on an atom far from the defect.[S11,S12] The final term E^q_{corr} corrects for the finite-size effect due to the long-range Coulomb interaction.

The calculations of the chemical potential μ, potential alignment ΔV, finite size correction E^q_{corr}, and intrinsic Fermi level $E_F^{(i)}$ are discussed in detail in the following.

We neglect the vibrational contributions to the DFEs, and the effect of thermal expansion on the DFEs, as these are typically small in the present compounds.[S7,S13]

3.1 Chemical potential

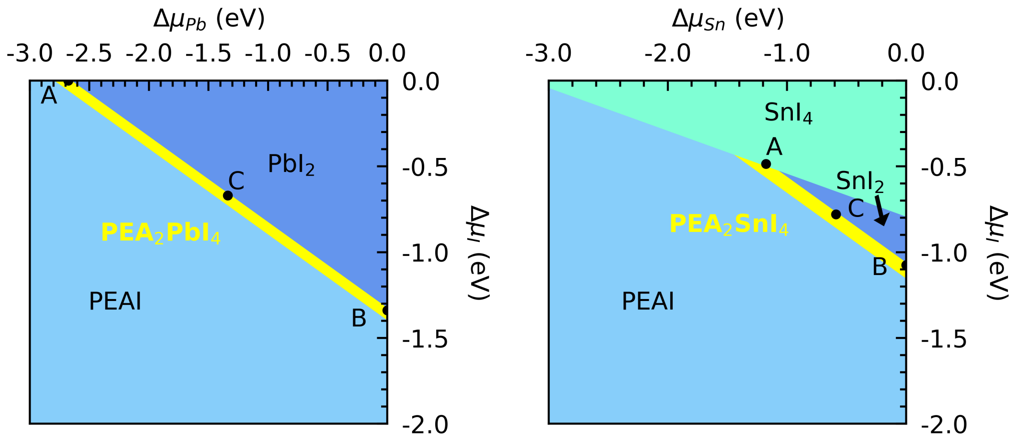

Figure S1: Calculated stability diagrams of PEA_2PbI_4 and PEA_2SnI_4. $\mu_I = \mu_{I_2,\text{molecule}}/2$ corresponds to $\Delta\mu_I = 0$ in the figures, and $\mu_M = \mu_{M,\text{bulk}}$ defines $\Delta\mu_M = 0$ for M = Pb, Sn. The points A and B define iodine-rich and iodine-poor conditions, respectively. Iodine-medium conditions (point C) are defined as halfway between points A and B.

Taking PEA_2PbI_4 for example, the chemical potentials μ_i of atomic species i are calculated by assuming that the perovskite is stable, so using $2\mu_{\text{PEA}} + \mu_{\text{Pb}} + 4\mu_{\text{X}} = \mu_{\text{PEA}_2\text{PbI}_4}$ as a constraint, where for $\mu_{\text{PEA}_2\text{PbI}_4}$ we use the DFT total energy per formula unit of the PEA_2PbI_4 perovskite. Furthermore, we assume that the perovskite is in equilibrium with the PbI_2 phase, so $\mu_{\text{Pb}} + 2\mu_{\text{I}} = \mu_{\text{PbI}_2}$, with μ_{PbI_2} the DFT total energy per formula unit of PbI_2. All μ_i can now be expressed in terms of a single parameter, μ_I, which is constrained by $\mu_{\text{PbI}_2} - \mu_{\text{Pb,bulk}} \leq 2\mu_{\text{I}} \leq \mu_{\text{I}_2,\text{molecule}}$. The outer bounds define I-poor (or Pb-rich) or I-rich conditions, respectively, with $\mu_{\text{Pb,bulk}}$ and $\mu_{\text{I}_2,\text{molecule}}$ the DFT total energies of bulk Pb metal, and an I_2 molecule. I-poor and I-rich conditions are indicated by points B and A, respectively, in Figure S1(a). The I-medium condition (point C) is defined as the halfway between points A and B, which is the focus of this work. The stability diagram of BA_2PbI_4 is similar to PEA_2PbI_4. For PEA_2SnI_4, the allowed interval for μ_I has to be narrowed down to prevent

the formation of other phases, such as SnI_4,[S14–S16] see Figure S1(b). For $PEA_2Sn_{0.5}Pb_{0.5}I_4$, we take the point C in the stability diagram of PEA_2SnI_4 to get the μ_I and μ_{Sn}. With this μ_I, the μ_{Pb} is then determined in Figure S1(a).

3.2 Finite-size supercell correction and potential alignment

The correction scheme for the finite-size effect proposed by Freysoldt, Neugebauer, and Van de Walle (FNV) is adopted.[S17] The correction energy is expressed as

$$E_{\text{corr}}^{\text{FNV}} = E_{\text{lat}} - q\Delta V_{q/b}, \tag{S2}$$

where the E_{lat} is the lattice energy induced by the electrostatic interaction between periodically repeated images of the charged defect, and $q\Delta V_{q/b}$ aligns the electrostatic potential of the charged defective supercell with respect to that of the pristine bulk supercell. As the FNV approach incorporates the potential alignment, the Delta V term in Equation 1 then should be omitted.[S12]

The lattice energy E_{lat} is inversely proportional to the dielectric constant ε. The high frequency (ε_∞) and static (ε_0) dielectric constants of each 2D perovskite, calculated using the functional PBE, and the lattice energies are shown in Table S2. As suggested in Ref. S12, the static dielectric constant (ε_0) is used in the correction scheme, where the ionic contribution is calculated using density functional perturbation theory.

Table S2: High frequency (ε_∞) and static dielectric (ε_0) constants of each 2D perovskite, calculated at the PBE level, and the resulting lattice energies (E_{lat}). The dielectric tensors are provided as a 3×3 matrix row by row in the order ε_{xx}, ε_{xy}, ε_{xz}, ε_{yx}, ε_{yy}, ε_{yz}, ε_{zx}, ε_{zy}, ε_{zz}. The lattice energy depends on the charge states (q) of defects.

Perovskite	ε_∞			ε_0			E_{Lat} (eV)
	3.91	0.02	-0.09	7.62	0.32	-0.08	0.10 ($q = \pm1$)
PEA_2PbI_4	0.02	3.91	-0.10	0.32	8.04	-0.14	0.38 ($q = \pm2$)
	-0.09	-0.10	3.13	-0.08	-0.14	4.46	
	3.77	0.00	0.00	8.91	0.00	0.00	0.11 ($q = \pm1$)
BA_2PbI_4	0.00	3.73	0.00	0.00	7.72	0.00	0.43 ($q = \pm2$)
	0.00	0.00	3.05	0.00	0.00	4.11	
	4.55	0.01	-0.10	11.55	0.29	-0.09	0.05 ($q = \pm1$)
PEA_2SnI_4	0.01	4.55	-0.10	0.29	11.00	0.07	0.21 ($q = \pm2$)
	-0.10	-0.10	3.20	-0.09	0.07	4.62	
	4.17	0.03	-0.10	9.05	0.28	0.11	0.07 ($q = \pm1$)
$PEA_2Pb_{0.5}Sn_{0.5}I_4$	0.03	4.17	-0.10	0.28	9.39	-0.11	0.30 ($q = \pm2$)
	-0.10	-0.10	3.16	0.11	-0.11	4.50	

3.3 Intrinsic Fermi level

The intrinsic Fermi level can be determined by the charge neutrality condition, which expresses the fact that, if no charges are injected in a material, it has to be charge neutral

$$p - n + \sum_{D^q} q\, c(D^q) = 0, \tag{S3}$$

where p and n are the charge densities of holes and electrons of the semiconductor material, $c(D^q)$ is the concentration of defect D^q, and the sum is over all types of charged defects. The concentrations can be calculated from Boltzmann statistics

$$c(D^q) = c_0(D^q) \exp\left[-\frac{\Delta H_f(D^q)}{k_B T}\right], \tag{S4}$$

where $c_0(D^q)$ is the density of possible sites for the defect (defined by the number of possible sites for the defect D^q in the unit volume), T is the temperature, k_B is the Boltzmann constant, and $\Delta H_f(D^q)$ follows from Equation S1. Obviously, p, n, and $c(D^q)$ are functions

of E_F, so the charge neutrality condition, Equation S3, serves to determine the intrinsic position of the Fermi level $E_F^{(i)}$.

The hole concentration p can be calculated from the density of states near the VBM

$$p = N_V^{2D} \frac{1}{l_z} \exp\left[-\frac{E_F}{k_B T}\right], \text{ where } N_V^{2D} = 2\frac{2\pi m_h^* k_B T}{h^2}. \tag{S5}$$

In Equation S5, N_V^{2D} is the density of states near the VBM, which we model assuming 2D isotropic parabolic dispersion of the bands in the metal-iodide plane, characterized by the effective hole mass m_h^*, and zero dispersion of the bands perpendicular to that plane. The m_h^* for PEA_2PbI_4, BA_2PbI_4 and PEA_2SnI_4, taken from Ref. S18, are $0.25m_0$, $0.39m_0$, and $0.15m_0$, respectively, where m_0 is the mass of the electron in vacuum. For $PEA_2Sn_{0.5}Pb_{0.5}I_4$ we use the same value as for PEA_2SnI_4. The N_v^{2D} is divided further by the thickness of one layer of the 2D perovskite, l_z, defining the 3D effective density of states.

In principle, the electron concentration n can be calculated along the same lines. However, it turns out that all materials considered here are intrinsically p-type doped. The intrinsic hole density p can be large and important for maintaining the charge neutrality together with the charged defects, while the intrinsic electron density n is negligibly small.

4 Charge state transition level

Under operating conditions, charges are injected in the material, shifting the positions of the (quasi) Fermi levels for electrons and holes. The charge state transition level (CSTL) $\varepsilon(q/q')$ is defined as the Fermi level position where the charge states q and q' of the same type of defect have equal formation energy, $\Delta H_f(D^q) = \Delta H_f(D^{q'})$. As the DFEs have a simple linear dependence on E_F, Equation S1, this condition can be expressed as

$$\varepsilon(q/q') = \frac{\Delta H_f(D^q, E_F = 0) - \Delta H_f(D^{q'}, E_F = 0)}{q' - q}, \tag{S6}$$

where $\Delta H_f(D^q, E_F = 0)$ is the DFE calculated at $E_F = 0$.

S2 Mixed Pb-Sn perovskite

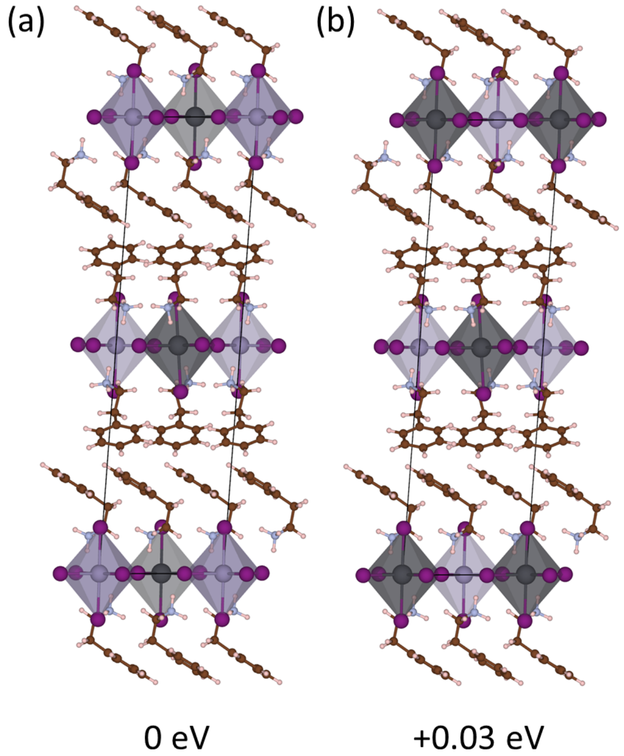

Figure S2: Optimized structures of two possible configurations of $PEA_2Pb_{0.5}Sn_{0.5}I_4$ with well mixed Pb and Sn.

The structure within the inorganic layers in 2D perovskites mainly determines the electronic structure, whereas the interaction between the layers is relatively less important. Therefore, we mix Pb and Sn within the same layer. Whereas the stacking pattern of adjacent layers has two possibilities, as shown in Figure S2, the second configuration is 0.03 eV per unitcell less stable than the first one. Therefore, we choose the one in Figure S2(a) as the model for studying defects. Another possible configuration can be one pure Sn layer stacked with one pure Pb layer, but there the local interaction is expected to be similar to the pure Sn- or Pb-based perovskites.

S3 DFEs at different growth conditions

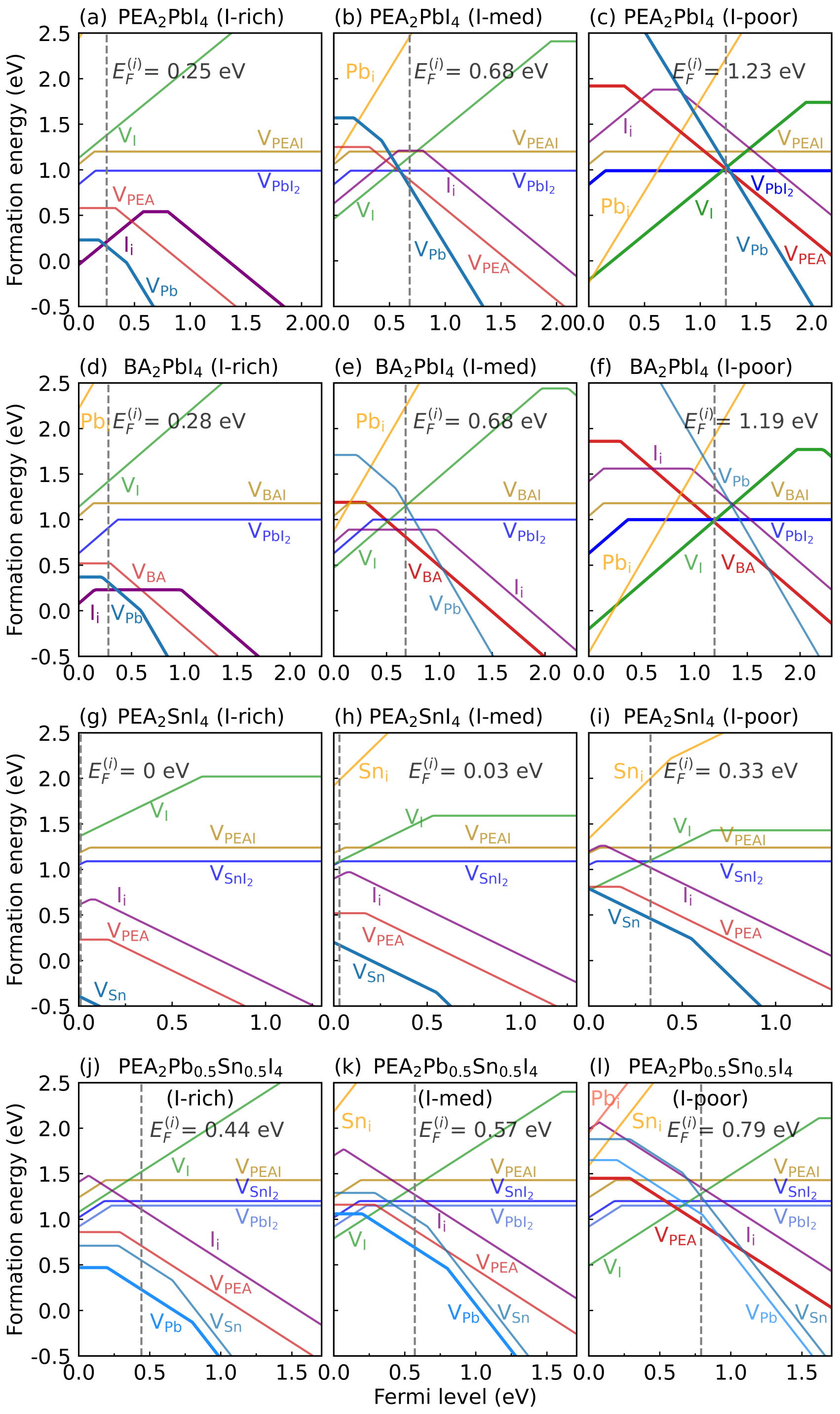

Figure S3: Defect formation energies in PEA_2PbI_4, BA_2PbI_4, PEA_2SnI_4, and $PEA_2Pb_{0.5}Sn_{0.5}I_4$ as function of the position of the Fermi level at different growth conditions; the intrinsic Fermi level is indicated by the grey dashed line.

References

(S1) Kresse, G.; Hafner, J. Ab initio molecular dynamics for liquid metals. *Phys. Rev. B* **1993**, *47*, 558–561.

(S2) Kresse, G.; Furthmüller, J. Efficient iterative schemes for ab initio total-energy calculations using a plane-wave basis set. *Phys. Rev. B* **1996**, *54*, 11169–11186.

(S3) Kresse, G.; Furthmüller, J. Efficiency of ab-initio total energy calculations for metals and semiconductors using a plane-wave basis set. *Comput. Mater. Sci.* **1996**, *6*, 15–50.

(S4) Peng, H.; Yang, Z.-H.; Perdew, J. P.; Sun, J. Versatile van der Waals Density Functional Based on a Meta-Generalized Gradient Approximation. *Phys. Rev. X* **2016**, *6*, 41005.

(S5) Sun, J.; Ruzsinszky, A.; Perdew, J. P. Strongly Constrained and Appropriately Normed Semilocal Density Functional. *Phys. Rev. Lett.* **2015**, *115*, 36402.

(S6) Sabatini, R.; Gorni, T.; de Gironcoli, S. Nonlocal van der Waals density functional made simple and efficient. *Phys. Rev. B* **2013**, *87*, 041108.

(S7) Xue, H.; Brocks, G.; Tao, S. First-principles calculations of defects in metal halide perovskites: A performance comparison of density functionals. *Phys. Rev. Mater.* **2021**, *5*, 125408.

(S8) Du, K.-z.; Tu, Q.; Zhang, X.; Han, Q.; Liu, J.; Zauscher, S.; Mitzi, D. B. Two-Dimensional Lead(II) Halide-Based Hybrid Perovskites Templated by Acene Alkylamines: Crystal Structures, Optical Properties, and Piezoelectricity. *Inorg. Chem.* **2017**, *56*, 9291–9302.

(S9) Billing, D. G.; Lemmerer, A. Synthesis, characterization and phase transitions in the inorganic-organic layered perovskite-type hybrids $(C_nH_{2n+1}NH_3)_2PbI_4$, n = 4, 5 and 6. *Acta Crystallogr. Sect. B Struct. Sci.* **2007**, *63*, 735–747.

(S10) Gao, Y.; Wei, Z.; Yoo, P.; Shi, E.; Zeller, M.; Zhu, C.; Liao, P.; Dou, L. Highly Stable Lead-Free Perovskite Field-Effect Transistors Incorporating Linear Π-Conjugated Organic Ligands. *J. Am. Chem. Soc.* **2019**, *141*, 15577–15585.

(S11) de Walle, C. G. V.; Neugebauer, J. First-principles calculations for defects and impurities: Applications to III-nitrides. *J. Appl. Phys.* **2004**, *95*, 3851–3879.

(S12) Komsa, H. P.; Rantala, T. T.; Pasquarello, A. Finite-size supercell correction schemes for charged defect calculations. *Phys. Rev. B* **2012**, *86*, 045112.

(S13) Wiktor, J.; Rothlisberger, U.; Pasquarello, A. Predictive Determination of Band Gaps of Inorganic Halide Perovskites. *J. Phys. Chem. Lett.* **2017**, *8*, 5507–5512.

(S14) Shi, T.; Zhang, H.-S.; Meng, W.; Teng, Q.; Liu, M.; Yang, X.; Yan, Y.; Yip, H.-L.; Zhao, Y.-J. Effects of organic cations on the defect physics of tin halide perovskites. *J. Mater. Chem. A* **2017**, *5*, 15124–15129.

(S15) Meggiolaro, D.; Ricciarelli, D.; Alasmari, A. A.; Alasmary, F. A. S.; De Angelis, F. Tin versus Lead Redox Chemistry Modulates Charge Trapping and Self-Doping in Tin/Lead Iodide Perovskites. *J. Phys. Chem. Lett.* **2020**, *11*, 3546–3556.

(S16) Xue, H.; Brocks, G.; Tao, S. Intrinsic defects in primary halide perovskites: A first-principles study of the thermodynamic trends. *Phys. Rev. Mater.* **2022**, *6*, 055402.

(S17) Freysoldt, C.; Neugebauer, J.; Van de Walle, C. G. Fully Ab Initio Finite-Size Corrections for Charged-Defect Supercell Calculations. *Phys. Rev. Lett.* **2009**, *102*, 16402.

(S18) Dyksik, M.; Duim, H.; Zhu, X.; Yang, Z.; Gen, M.; Kohama, Y.; Adjokatse, S.; Maude, D. K.; Loi, M. A.; Egger, D. A.; Baranowski, M.; Plochocka, P. Broad Tunability of Carrier Effective Masses in Two-Dimensional Halide Perovskites. *ACS Energy Lett.* **2020**, *5*, 3609–3616.
